# Molecular Mimics: How Viral Genomes Dupe Their Host by Usurping CTCF to Establish Infection

**DOI:** 10.3390/v18040456

**Published:** 2026-04-10

**Authors:** Clairine I. S. Larsen, Rhiannon R. Abrahams, Kinjal Majumder

**Affiliations:** 1 Cellular and Molecular Biology Graduate Program, University of Wisconsin-Madison, Madison, WI 53706, USA; cilarsen@wisc.edu; 2 Institute for Molecular Virology and McArdle Laboratory for Cancer Research, University of Wisconsin, Madison, WI 53706, USA; rrabrahams@wisc.edu; 3 Microbiology Doctoral Training Program, University of Wisconsin-Madison, Madison, WI 53706, USA

**Keywords:** chromosome conformation capture, DNA viruses, CTCF, insulator, gene regulation

## Abstract

The eukaryotic genome is organized into distinct structural units dictated by architectural proteins. The major host architectural protein CCCTC-binding factor (CTCF) is usurped by DNA viruses to regulate viral gene expression. This review will discuss the major ways large (EBV, HSV, HCMV) and small (HPV, HBV, AAV) DNA viruses mimic eukaryotic genome topology using CTCF to regulate viral gene expression. We will further discuss how changes in genome topology can drive virally induced oncogenic progression. Knowledge gained from studying viral genome folding mechanisms will inform the development of targeted anti-viral agents and inform the modification of viruses to serve as gene therapy vectors.

## 1. Introduction

As obligate intracellular pathogens, viruses utilize host proteins to establish and replicate themselves within the cellular milieu. Consequently, key drivers of cellular gene expression, such as the regulation of the chromatin landscape and genome organization by the host protein CCCTC-binding factor (CTCF), are also appropriated by the virus. CTCF’s role in transcriptional regulation, genome stability, RNA binding and splicing of the eukaryotic genome have been well characterized and reviewed elsewhere [1,2,3]. CTCF primarily binds a consensus sequence on DNA molecules, making the viral genome that contain cognate motifs an excellent target for this association. In addition to its topological regulation role, CTCF is also associated with the cellular DNA damage response (DDR) [4]. CTCF rapidly associates with sites of DNA damage to facilitate homologous recombination (HR) repair [5,6]. Additionally, CTCF’s architectural role controls the impact of DNA damage by restricting the spread of phosphorylated H2AX histones [7,8]. DNA viruses recruit, suppress, and activate numerous DNA damage response proteins to regulate their lifecycle, presenting CTCF as a key protein at the intersection of DDR and chromatin regulation for viral manipulation [9,10,11].

Recent findings have identified cellular transduction pathways, such as those activated by DDR signaling, that also recruit CTCF and are known to regulate viral infection. These phenomena have been elegantly described in multiple reviews [9,12,13,14,15]. Continuing evidence has highlighted the complex and essential role played by CTCF in regulating the life cycle of DNA viruses (both large and small) and retroviruses. However, it remains unknown whether CTCF binding is the principal driver of changes in viral chromatin landscape or whether post-translational modifications associated with changes in the cellular environment regulate CTCF’s local function.

CTCF is a versatile evolutionarily conserved host protein that has been well characterized for its role in regulating host chromatin organization and gene expression [16,17]. Structurally, CTCF contains 11 zinc finger domains that drive the recognition and asymmetric binding to its cognate DNA motifs (referred to as CTCF binding element, CBE) [18,19,20,21]. The central ZnF domains are flanked by disordered C- and N-terminal domains that drive protein–protein interactions [18,22]. The C-terminal region of dimerized CTCFs interacts with components of the Cohesin complex (SMC1, SMC3, RAD21, and STAG1/2) and is vital for stabilizing chromatin loops (using loop extrusion mechanism, described below) to regulate host gene expression [23,24,25]. Additionally, CTCF interacts with transcriptional regulators (e.g., YY1, RNA Pol II) and chromatin modifiers (PRC2 and histone deacetylase SIN3) [26,27,28,29,30,31]. Post-transcriptional modifications mediate CTCF functions such as: (1) poly(ADP-ribosylation) by PARP at the N-terminal end to facilitate insulator and regulation of rRNA and barrier function of CTCF [32,33]; (2) sumoylation at the N- and C-terminal regions have been associated with repressive functions of c-myc and colocalization with the Polycomb Repressive Complex, PRC2 [34]; and (3) phosphorylation across the zinc finger domains regulate CTCF DNA binding and phosphorylation by CK2 facilitates activation of gene expression [35,36]. These observations have begun to illuminate the complexity of the cellular signaling pathways that must be activated to facilitate CTCF function. Since viruses are known to modulate cellular pathways such as the DNA Damage Response pathway [9,10,12,37], it is likely that these signals shape the local activation of CTCF to direct viral gene regulation.

In this review we will discuss the molecular mimicry deployed by DNA viruses to recruit and usurp the multiple functions of CTCF that favor viral life cycle.

## 2. Large DNA Viruses and Host Architectural Proteins

### 2.1. Herpesvirus Genomes Mimic Cellular TADs

**Figure 1:**
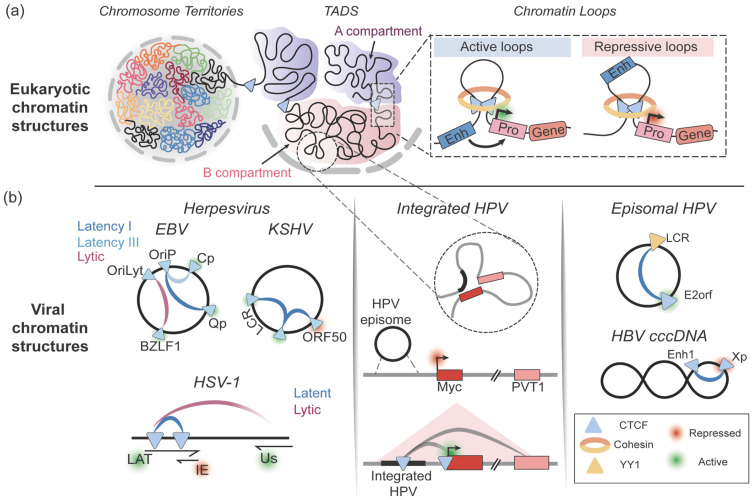
Viruses utilize CTCF to regulate the viral genome by mimicking host topological structures. (**a**) Schematic representation of the levels of eukaryotic chromosome organization driven by CTCF. Chromosomes occupy distinct territories and are subdivided into independently packaged *cis*-interacting Topologically Associating Domains (TADs) of transcriptionally active (A compartment) and repressive (B compartment) chromatin. Individual chromatin loops regulate promoter–enhancer interactions. (**b**) Schematic representation of the key CTCF binding sites in DNA viruses regulating viral gene expression. *Cis* interactions are shown by connecting lines. Blue lines indicate interactions during latency; red lines indicate interactions during lytic infection.

The eukaryotic genome is organized into Topologically Associating Domains (TADs; Figure 1a) that serve as functional units for gene regulation by separating genomic regions packaged in active chromatin from those in inactive chromatin [38,39,40]. The fundamental structural unit of a TAD is a DNA loop [3]. These genomic loops are categorized as structural (aimed at DNA packaging, described below) and functional DNA loops (that form promoter–enhancer contacts). Functional loops correlate with the transcriptional state of the genes that make up a TAD. Structural loops exist on a large topological scale (100–1500 kb) to restrict genome packaging to *cis*-interactions using the nuclear matrix or chromosome scaffold as the anchor [41]. A defining feature of TADs is the enrichment of CTCF at the boundaries where it acts as a chromatin barrier element (referred to as an insulator) [42,43]. These DNA loops are formed by extrusion of the genome through a collar-like structure generated by Cohesin proteins until distally bound CTCF elements are brought together, a phenomenon referred to as the loop extrusion [24]. Transcriptionally active domains contain smaller functional loops that are facilitated by CTCF binding and stabilized by Cohesin or Mediator complexes [44,45]. Importantly, the boundaries of these TAD borders and their internal structures are highly dynamic, likely driven by the stability of CTCF binding which is subjected to temporal regulation caused by DNA damage and cell cycle [46,47]. Since large DNA viruses like KSHV, EBV, and HSV-1 possess genomes that are approximately 100–170 kb in size (roughly the same size as a TAD), they have evolved mechanisms to usurp the folding principles of the eukaryotic genome to mimic a TAD (Figure 1b, left columns).

#### 2.1.1. Gammaherpesviruses

Upon entering the host cell, EBV and KSHV deliver linear dsDNA genomes that are rapidly circularized and decorated with cellular histones so that they begin to resemble host chromosomes [48,49]. The success of long-term gammaherpesvirus infections depend on the establishment of latency to avoid immune detection and lifelong maintenance [48,49]. A tightly regulated switch inactivates persistence mechanisms to engage lytic gene expression, and improper timing of this transition is detrimental to the virus. The mechanisms that regulate the establishment of latency and latent-to-lytic switch in EBV and KSHV life cycles are regulated by multiple CTCF loops that organize chromatin boundaries between latent and lytic genes.

The EBV genome contains at least 17 CBEs, many of which had initially been identified as DNase I hypersensitive sites by footprinting assays [50,51,52]. During the latent phase of EBV life cycle, CTCF binding is enriched at the viral promoters Cp, Qp, EBERs and Wp in the Latency Control Region (LCR) [51]. Unlike KSHV, EBV has a dynamic latency program with three different latency types characterized by differences in gene expression. Latency I is the most restricted with only EBNA1 and some long non-coding RNAs being expressed. In contrast, latency III is characterized by the expression of all EBNA proteins, non-coding RNAs, and LMP1/2; observed in proliferating B cells upon EBV infection and in DLBCL lymphomas [48]. CTCF serves as a critical regulator of these latency programs by facilitating the formation of a chromatin loop between OriP and Qp during latency I [53]. In contrast, latency III is defined by a spatial interaction between OriP and Cp [52,54]. These 3D conformations associated with latency aid in the expression of latent genes and repression of lytic genes as shown by the colocalization of RNA Pol II and CTCF [55]. Additionally, these CTCF-bound sites are enriched in Cohesin [55], and disruption of CTCF or Cohesin results in a loss of *cis* interactions [52,56,57]. These observations suggest that latency-associated loops on the EBV genome are likely to be established by the loop extrusion mechanism. In doing so, EBV has seemingly adopted the eukaryotic mechanism of compartmentalizing chromatin to create active (latent) and inactive (lytic) regions of the viral chromatin landscape, mimicking the structure of cellular TADs.

Bearing a resemblance to the dynamic nature of eukaryotic gene regulation within an individual TAD, CTCF also regulates the coordination of latency I vs. III genes within the larger active latent compartment. Histones are deposited along the EBV episome in a manner that resembles host chromatin [58]. This allows CTCF to deploy an additional (and perhaps redundant) mechanism of gene control by acting as an insulator element, serving as a chromatin barrier to regulate the epigenetic landscape of the latency-associated viral promoters. During latency I, the Qp region is enriched for post-translational histone marks associated with active chromatin (H3K4me3 and H4 acetylation) [53] while the latency III Cp promoter is depleted in active marks [59]. However, during latency III this chromatin profile is reversed, aiding in the suppression of Qp and activation of Cp [60]. Interestingly, neither of these promoters are enriched for repressive chromatin during latency [60,61]. Disruption of CTCF at either of these sites results in a dysregulation of loop formation and intrusion of repressive chromatin marks despite the latency stage [13,53]. This regulation of local chromatin landscape by CTCF-bound elements are likely loop-independent because overall CTCF occupancy at Qp and Cp does not drastically change [54]. Taken together, these exciting findings have shed light into the direction of causality that connects looping, chromatin landscape and viral pathogenesis.

While CTCF controls the dynamic latency programs and latent-to-lytic switch in EBV, it plays an insulator and stabilization role in the latent and lytic KSHV lifecycle (Figure 1). A total of 25 CBEs have been identified in KSHV with the majority of sites found in the Latency Control Region (LCR) and at the promoter of ORF50, which regulates the expression of the immediate early protein RTA [62]. Regulatory 3D interactions between ORF50 and the LCR; and the 5′ and 3′ ends of the LCR establish the latent program of KSHV [63,64]. Distinct from their role in regulating EBV conformation, CTCF sites on KSHV direct a conformation where latency genes are contained within a single loop that is isolated from lytic genes. CTCF binding at these sites colocalizes with Cohesin to regulate RNA PolII occupancy and latent transcription [65]. Cohesin’s interaction with CTCF seems to be dependent on cell cycle, suggesting another layer of control to CTCF-mediated regulation of KSHV [66]. There is some evidence that CTCF impacts the histone profile of KSHV by preventing excessive H3K4me3 enrichment [65]. However, whether this is a direct effect on the viral chromatin landscape or an indirect consequence of loop formation/dissolution by CTCF remains unknown.

#### 2.1.2. Alphaherpesviruses

Alphaherpesvirus HSV-1 genomes are 152 kb long and contain 7 CTCF binding elements [67]. Interestingly, rather than being regularly spaced throughout the genome, the CBEs on HSV-1 are concentrated in the Internal Repeat Short and Long regions (IR_S_ and IR_L_ respectively). Specifically, a few key CBEs (CTRL1/2, CTa’m, and CTRS1/2/3) are positioned flanking the LAT latency gene, and the immediate early lytic genes ICP0, and ICP4 [67,68,69]. The recent application of 4C-seq in the neuronal precursor cell line LUHMES showed that HSV-1 adopts a 3D conformation that is reorganized upon loss of CTCF binding at CTRL2 [70]. These interactomes show that HSV-1 forms short-range *cis*-loops within the Internal Repeat regions and long-range interactions with the Unique Short region (U_S_) and a putative novel CBE (Figure 1b, left panel). CTCF binding at CTRL2 regulates expression of the overlapping LAT and ICP0 genes, maintaining latency through tight control of the boundary between repressive ICP0 and active LAT histone marks [71]. CTCF is evicted at the CTRL2 region during reactivation and is necessary for the expression of lytic genes. Loss of CTCF in latency results in shedding of infectious virus and uncontrolled ICP0 expression [72,73]. However, these observations contrast with findings that demonstrated that loss of CTRL2 during lytic infection led to a loss of IE gene expression and an increase in repressive histone marks (H3K9me3 and H3K27me3) [74]. A role for CTCF in lytic infection is further supported by a loss of binding leading to a decrease in viral transcription, viral genome copy-number and virus yield [75]. Therefore, it remains possible that the eviction of CTCF at the latent-lytic transition is needed but CTCF binding is re-established with a new role of promoting lytic gene expression. This hypothesis is supported by the similar findings in EBV, where CTCF-mediated control of latent gene expression is carried out by CTCF-eviction early in reactivation but is re-deposited at the same CBEs 24 h into lytic infection and is also necessary for the lytic lifecycle [55,76].

#### 2.1.3. Betaherpesviruses

The Betaherpesvirus Human Cytomegalovirus (HCMV) has a 235 kb genome but only two CBEs have been identified [77]. One of these sites, on the major lytic promoter named MIEP, forms a repressive local loop with the enhancer to downregulate MIE gene expression and maintain latency [78,79]. Interestingly, these two CTCF elements are in a convergent orientation, which has been proposed to be favorable for loop formation across the human genome that lead to the establishment of TADs globally [77].

### 2.2. Regulation of Architectural Proteins by Local Post-Translational Modifications (PTMs)

The ubiquitous nature of CTCF binding in both the host and large DNA viruses is in contradiction with the fine-tuning required for focused gene expression. This suggests that additional controls are needed to regulate CTCF function at distinct times. One such level of control is in the form of post-translational modifications (PTMs). CTCF regulation by PTM enzymes like PARP, CK2, LATS, SUMO, and PLK1 have been well studied in the context of the host. However, in viral systems only the role of PARP in regulating CTCF-mediated control of EBV life cycle has been investigated [33,34,46,54,80,81,82,83].

The major cellular DDR protein PARP1 has recently been identified as a key regulator of CTCF function, with CTCF having a feedback role on PARP1 activity [4]. CTCF acts on PARP1 to inhibit DNA methylation at CBEs, facilitating CTCF binding [84]. On the other hand, CTCF can undergo post-translational modification by PARP1, referred to as PARylation, at the N terminus to regulate CTCF activity [32,33]. During EBV infection, high levels of PARP activity are detected during the latency III program [85]. This correlates with the stabilization of CTCF binding to the Cp via PARylation [54]. However, CTCF binding at Qp is not regulated by PARP activity. This suggests a model in which the increase in global PARP levels regulate the latency I/III switch. Interestingly, CTCF and multiple nuclear architectural proteins are associated with nascent EBV DNA during virus replication, suggesting a role in lytic infection [86]. CTCF and Cohesin colocalize with the EBV immediate-early gene BZLF1 to regulate expression of Zta protein. During latency Myc binds to oriLyt; however, upon reactivation signals, such as differentiation, a decrease in Myc abundance allows for a conformational change in EBV 3D structure and the formation of a loop between BZLF1 and oriLyt [87]. Interestingly, an earlier study showed that along with CTCF, PARP binds to BZLF1 to restrict reactivation. Like its role in regulating CTCF at Cp, a loss of PARP activity destabilizes CTCF binding [76]. Together, these individual observations hint at a model in which CTCF regulates both latent and lytic gene expression profiles in EBV but requires PARP-mediated modifications to license its function. The stepwise regulation of what confers PARP-mediated looping properties to individual CTCF sites remains largely unknown. The finding that PARP regulates CTCF on EBV suggests that other PTMs can act as another layer of control on CTCF in the viral context.

## 3. Small DNA Viruses and Host Architectural Proteins

### 3.1. Human Papillomavirus Tethering and Integration

The best characterized example of CTCF interaction with small DNA viruses is in the ~8 kb Human Papilloma Viruses (HPV) [14,88,89,90]. The HPV family is composed of many HPV types and can be divided into low-risk HPVs (such as 11 and 6b) that cause warts and high-risk HPVs (such as 16,18 and 31) that lead to cervical, anogenital and head and neck cancers. A defining feature of the high-risk HPVs is their ability to interact with CTCF at specific sites to regulate oncogene expression and maintain an extrachromosomal episome [88]. Two major regions of CTCF binding are found on the genomes of the high-risk HPVs (Figure 1b, middle panel). CBEs in the late gene region (which are also found in low-risk HPVs) regulate the differentiation-dependent amplification of HPV31 [91]. Loss of CpG methylation during differentiation allows CTCF to bind and direct late gene expression [92,93]. Specific to high-risk HPVs, CTCF binding to E2orf region controls oncogene expression (E6/E7) and disruption of binding causes uncontrolled early expression and cellular hyperproliferation [88]. At the cellular and organismal level, expression of these oncogenes can contribute to the formation of pre-cancerous lesions and malignancies.

In the eukaryotic genome, CTCF facilitates the formation and stability of short-range functional loops to control gene expression. Given the smaller size of HPV and the presence of CTCF binding elements, it is plausible that HPV has evolved to utilize CTCF to mimic the short-range looping seen on the eukaryotic genome (Figure 1b, middle panel). In eukaryotic genomes these short-range loops are mostly formed via dimerization of two distally bound CTCF proteins that extrude the intervening DNA through the Cohesin loops (loop extrusion) [24,94]. While this mechanism is conserved in large DNA viruses, HPV 3D conformation is additionally regulated by interactions between CTCF and the cellular transcription factor YY1 [89]. A *cis* interaction loop is formed between CTCF bound at E2orf and YY1 bound at the Upstream Regulatory Region (URR) and is stabilized by Cohesin [89]. Interestingly, the Cohesin subunit SMC1 is phosphorylated by CHK2 in the ATM signaling pathway, further demonstrating the interplay between virus-induced DDR signals and 3D conformation [91]. This loop facilitates the suppression of E6/E7 expression through the recruitment of the Polycomb Repressor Complex (PRC) resulting in trimethylation of histone H3 at lysine 27 [89]. Upon differentiation this loop is lost through a downregulation of cellular YY1 by the HPV early oncogene expression resulting in E6/E7 expression [89]. These observations suggest that there is either a cell-cycle dependent or DDR-dependent role in regulating CTCF-mediated loop formation.

Dissecting the role of CTCF in HPV life cycle is further complicated by the integration of the viral genome into the host at cellular super-enhancer regions, many of which are fragile sites and have significant clusters of CTCF/Cohesin protein binding [95,96,97]. Integration of the HPV genome introduces an additional CTCF site into the host genome, which is comparable with HTLV1 inserting a CTCF binding site into the human genome, disrupting the local 3D chromatin environment [98]. This is shown by increased chromatin accessibility within 100 kb of HPV integration sites inducing differential gene expression profiles in HPV-positive tumors [99]. Particularly, HPV integration occurs near transcriptional regulatory hubs associated with cellular oncogenes Myc and TP63 that cause cervical cancers [96]. The addition of these HPV CTCF sites facilitates a novel loop ~500 kb loop to the MYC/PVT1 regions resulting in oncogene upregulation that drives cancer progression [100]. Similarly, CBEs on the HTLV genome play a role in persistent infection, activation and expansion of T lymphocytes [101,102].

### 3.2. CTCF-Mediated Regulation of RNA Processing

In the eukaryotic genome CTCF binding can impact alternative splicing through stalling of Pol II to promote the use of weak splicing donor and acceptor sites [82]. This function of CTCF is adopted by multiple viruses including HPV, MVM, and HBV. These highly compact viruses rely on alternative splicing, multiple promoters and overlapping open reading frames to express multiple viral genes from a small genome. In HPV, loss of CTCF binding leads to an increase in unspliced products [90]. In contrast, in the episome form of HBV, known as covalently closed circular DNA (cccDNA), CTCF binds at Enhancer I and downstream transcriptional start site Xp to generate a repressive loop (Figure 1b, right panel). Loss of CTCF binding at two sites within Enhancer I results in an increase in spliced transcripts correlating with changes in nucleosome positioning [103,104]. These roles of CTCF extend beyond regulation of oncogenic viruses. In the autonomous parvovirus Minute Virus of Mice (MVM), CTCF binding is essential for splicing of the MVM small intron and regulates the ratio of R2:R1 transcripts that yield the essential non-structural viral protein NS1 [105]. Absence of adequate levels of NS1 attenuates virus replication [106]. This phenomenon in small DNA viruses suggests the existence of evolutionarily conserved mechanisms where CTCF’s ability to regulate splicing on the host genome is usurped to aid in the expression of viral genes from small genomes.

## 4. Leveraging CTCF-Mediated Molecular Mimicry to Improve Gene Therapy

### 4.1. Adenovirus

The mechanistic dissection of how CTCF regulated gene expression across many DNA viruses has spurred the development of vector platforms that use CTCF binding elements to improve viral gene therapy. A prominent example of this is with Adenovirus (AdV), which has a 35 kb genome and has adapted CTCF-mediated chromatin regulation during late stage infection. While the mechanism of CTCF-dependent regulation of AdV is not entirely understood, binding of CTCF to the viral genome is dependent on viral DNA replication and enhances late gene expression [107,108]. Surprisingly however, a recent study identified the CTCF partner TFII-I as a viral restriction factor for AdV life cycle [109]. This suggests that CTCF’s ability to recruit activators and repressors of viral gene expression might vary temporally according to the stage of the viral life cycle and by signaling pathways activated by the virus (both locally and globally). These findings have contributed to improvements in engineering Adenoviral vectors.

The use of viral gene therapy platforms has been transformative in medicine to deliver functional gene copies, inactivate, or edit the host genome. Major gene therapy platforms have been developed using lentivirus, Adenovirus, and most recently Adeno-associated virus where in all cases some or all viral genes and regulatory elements are replaced with a therapeutic transgene driven by synthetic *cis*-elements [110,111]. CTCF has been leveraged in gene therapy to enhance expression, production and the safety of gene therapy vectors (described below). Current work has primarily utilized the insulator function of CTCF. In the integrative lentiviral gene therapies, CTCF has been added to ensure integration does not disrupt global host 3D conformation and expression [88,89,90]. Further the Adenovirus gene therapy vector was modified to contain CTCF binding elements at the 5′, 3′, or both 5′ and 3′ of a CMV-GFP cassette retaining viral genes as an insulator, and suppressed immune activation and prolonged GFP expression in mice [112].

### 4.2. Adeno-Associated Viruses

Adeno-associated virus (AAV) has emerged as a leading gene therapy platform largely because it does not integrate into the host genome and the ability to remove all viral genes enhances the safety of these vectors [113]. The addition of CTCF into the backbone of the AAV expression plasmid has been leveraged to decrease the cross-packaging of toxic DNA contaminates during AAV gene therapy vector production [114]. However, the mechanism by which CTCF enhances these processes have not been determined. These observations raise the question of whether CTCF regulates parvovirus lifecycles. Interestingly, proteomics studies in canine parvovirus (CPV) have demonstrated that the viral NS2 protein associates with host proteins involved in chromatin organization, including CTCF [115]. Consistent with these findings, our lab has recently identified using iPOND analysis that AAV mono-infection leads to replication stress and CTCF is found at AAV induced stalled cellular replication forks [116]. Additionally, affinity-tagged purification and imaging studies have identified two major PTM proteins of CTCF, PARP1 and the SMC2 protein of the Cohesin complex, associated with AAV non-structural proteins Rep68/78 [117]. We have further identified a role for PARylation in the establishment of AAV infection [118]. While a direct role for CTCF in regulating AAV has not been identified yet, these findings suggest that just like other DNA viruses big and small, AAV may also mimic the host genome by deploying the CTCF protein for the purpose of gene regulation.

## 5. Conclusions and Future Directions

The ability of CTCF to perform multifunctional roles that regulate how the cellular DNA gets packaged, expressed and repaired within the nucleus seems to be an attractive mechanism for viruses to usurp for their success. As shown in Table 1, large DNA viruses (particularly gammaherpesviruses) use CTCF to spatially isolate latent from lytic genes. However, small DNA viruses utilize CTCF for regulating short-range repressive loops or RNA processing. Despite intense studies and exciting discoveries over the last two decades on how viral genomes adopt looped conformations and how these structures are maintained by architectural proteins, much more remains to be mechanistically dissected:

• How is CTCF altered by post-translational modifications so that it can regulate latent, latent-to-lytic and lytic gene expression programs?
• What is the connection between the looping and chromatin insulator function of CTCF?
• Is the differential role of CTCF driven by its protein interaction partners?
• How is CTCF regulated by virus-induced DNA damage and virally induced alterations to the host cell cycle?
• How does CTCF-mediated regulation of gene expression and genome stability impact oncogenic progression?
• How can the functions of CTCF be leveraged for therapeutically beneficial applications?

A deeper understanding of how viruses deploy molecular mimicry using CTCF will help to improve detection modalities, uncover additional layers of viral signaling and engineer targetable viruses/vectors for efficient therapeutic interventions.

## Figures and Tables

**Table 1:** Comparative overview of the role of CTCF in DNA viruses. This table highlights the number of CBEs found through both in silico and in vivo methods, key locations discussed in this review, and a general function of CTCF binding in viral lifecycle and expression control.

Virus	Genome Size (kb)	Number of CTCF Sites	Key Locations/Regions	Function
EBV	170	17	OriP, Cp, Qp, OriLyt, BZLF1	Dynamic latency control, reactivation
KSHV	140	25	LCR, ORF50	Stable latency control
HSV-1	152	7	LAT, IE	Latency control, reactivation
HCMV	235	2	MIE	Lytic suppression
HPV	8	6–11	E2orf	Oncogene suppression, splicing
HBV	3.2	2	Enhancer I	Transcription, splicing
MVM	5	2	NS, VP	Splicing
AdV	35	3	Major late promoter, Hexon, E4orf	Replication

## Data Availability

No new data were created or analyzed in this study. Data sharing is not applicable to this article.

## Abbreviations

The following abbreviations are used in this manuscript:

3D	Three-dimensional
4C-seq	Circular chromosome conformation capture sequencing
AAV	Adeno-associated virus
AdV	Adenovirus
BZLF1	BamHI Z fragment leftward open reading frame 1
CBE	CTCF binding element
CK2	Casein kinase 2
CMV	Cytomegalovirus
Cp	C promoter
CPV	Canine parvovirus
CTCF	CCCTC-binding factor
DDR	DNA damage response
DLBCL	Diffuse large B-cell lymphoma
EBERs	Epstein–Barr virus-encoded small RNAs
EBNA1	Epstein–Barr virus nuclear antigen
EBV	Epstein–Barr virus
GFP	Green fluorescent protein
H3K27me3	Trimethylation of lysine 27 on histone 3
H3K4me3	Trimethylation of lysine 4 on histone 3
H3K9me3	Trimethylation of lysine 9 on histone 3
HBV	Hepatitis B virus
HCMV	Human cytomegalovirus
HPV	Human papillomavirus
HR	Homologous recombination
HSV	Herpes simplex virus
HTLV	Human T-lymphotropic virus
ICP0/4	Infected cell protein 0/4
IE	Immediate early
iPOND	Isolation of proteins on nascent DNA
IR	Internal repeat
KSHV	Kaposi’s sarcoma associated herpesvirus
LAT	Latency-associated transcript
LCR	Latency Control Region of EBV
LUHMES	Lund human mesencephalic cells
MIE	Major Immediate Early
MIEP	Major immediate-early promoter
MVM	Minute Virus of Mice
Myc	Myelocytomatosis
NS1/NS2	Non-structural protein 1/2
ORF	Open reading frame
OriP	Origin of plasmid replication
PARP/PARP1	Poly (ADP-ribose) polymerase
PVT1	Plasmacytoma Variant Translocation 1
Qp	Q promoter
RNA Pol II	RNA polymerase II
rRNA	Ribosomal RNA
SMC1/3	Structural maintenance of chromosomes protein
STAG1/2	Stromal antigen protein
TAD	Topological associating domain
TP63	Tumor protein p63
Wp	W promoter
YY1	Ying Yang 1
